# Desempenho dos Índices de Gravidade na Predição de Complicações Pós-Operatórias de Revascularização Miocárdica

**DOI:** 10.36660/abc.20190120

**Published:** 2020-09-18

**Authors:** Silvana Alves dos Santos Franzotti, Dyenily Alessi Sloboda, Juliana Rosendo Silva, Ellian Amorim Santos Souza, Jessica Zamora Reboreda, Renata Eloah de Lucena Ferretti-Rebustini, Lilia de Souza Nogueira

**Affiliations:** 1 Universidade de São Paulo Escola de Enfermagem São Paulo SP Brasil Universidade de São Paulo - Escola de Enfermagem, São Paulo, SP - Brasil; 2 Universidade de São Paulo Faculdade de Medicina Hospital das Clínicas São Paulo SP Brasil Universidade de São Paulo Faculdade de Medicina Hospital das Clínicas Instituto do Coração, São Paulo, SP – Brasil

**Keywords:** Doenças Cardiovasculares/complicações, Doenças Cardiovasculares/cirurgia, Revascularização Miocárdica/complicações, Cuidados Pós-Operatórios, Complicações Pós-Operatórias, Morbimortalidade, Indicadores de Gravidade de Doença, Unidade de Terapia Intensiva

## Abstract

**Fundamento:**

Os pacientes em pós-operatório (PO) de cirurgia de revascularização miocárdica (CRM) internados em unidade de terapia intensiva (UTI) apresentam risco de complicações que aumentam o tempo de permanência e a morbimortalidade. Portanto, é fundamental o reconhecimento precoce desses riscos para otimizar estratégias de prevenção e desfecho clínico satisfatório.

**Objetivo:**

Analisar o desempenho de índices de gravidade na predição de complicações em pacientes no PO de CRM durante a permanência na UTI.

**Métodos:**

Estudo transversal, com análise retrospectiva de prontuários eletrônicos de pacientes com idade ≥ 18 anos submetidos à CRM isolada e admitidos na UTI de um hospital cardiológico, em São Paulo, Brasil. As áreas sob as curvas *receiver operating characteristic* (AUC) com intervalo de confiança de 95% foram analisadas para verificar a acurácia dos índices *European System for Cardiac Operative Risk Evaluation* (EuroScore), *Acute Physiology and Chronic Health Evaluation* (APACHE II), *Simplified Acute Physiology Score* (SAPS II) e *Sequential Organ Failure Assessment* (SOFA) na predição de complicações.

**Resultados:**

A casuística foi composta por 366 pacientes (64,58±9,42 anos; 75,96% sexo masculino). As complicações identificadas foram respiratórias (24,32%), cardiológicas (19,95%), neurológicas (10,38%), hematológicas (10,38%), infecciosas (6,56%) e renais (3,55%). O APACHE II apresentou satisfatório desempenho para a predição de complicações neurológicas (AUC 0,72) e renais (AUC 0,78).

**Conclusão:**

O APACHE II se destacou na previsão das complicações neurológicas e renais. Nenhum dos índices teve bom desempenho na predição das outras complicações analisadas. Portanto, os índices de gravidade não devem ser utilizados indiscriminadamente com o objetivo de predizer todas as complicações frequentemente apresentadas por pacientes após CRM. (Arq Bras Cardiol. 2020; 115(3):452-459)

## Introdução

As doenças cardiovasculares são responsáveis por elevada taxa de mortalidade no Brasil e no mundo, com destaque à alta prevalência do infarto agudo do miocárdio (IAM).^1-3^ Dentre as principais terapias voltadas para o tratamento do IAM, aponta-se a cirurgia de revascularização miocárdica (CRM).^4,5^

Resultados bem-sucedidos da CRM dependem de um excelente cuidado pós-operatório (PO) realizado na unidade de terapia intensiva (UTI), uma vez que os pacientes estão expostos a inúmeros efeitos adversos decorrentes da complexa resposta inflamatória sistêmica orgânica, apresentam instabilidade hemodinâmica e maior risco de desenvolver complicações durante o tratamento intensivo.^6-9^

Reconhecer precocemente os pacientes que apresentam risco elevado de complicações é essencial para otimizar o tratamento, aumentar a chance de desfecho satisfatório,^6-9^ além de reduzir custos, uma vez a ocorrência destes eventos após CRM aumenta significativamente os gastos com tratamento e hospitalização.^10^

Estudos recentes têm mostrado a excelente acurácia de diferentes índices de gravidade na predição de mortalidade de pacientes no PO de cirurgia cardíaca na UTI.^11-13^Em relação à ocorrência de complicações, pesquisadores identificaram que o índice *European System for Cardiac Operative Risk Evaluation* (EuroScore) foi bom preditor de falência respiratória e lesão renal aguda dialítica em pacientes após cirurgia cardíaca.^14^

A partir do exposto, até o presente momento, não existem índices específicos capazes de prever satisfatoriamente o risco de um paciente desenvolver diferentes complicações na UTI após CRM. Além disso, na prática clínica, os profissionais intensivistas identificam estreita relação entre gravidade do paciente no PO de CRM e ocorrência de complicação.

Neste sentido, reforça-se a importância de analisar e investigar o desempenho de diferentes índices de gravidade na predição dessas complicações uma vez que essas informações poderão auxiliar em estratégias de tratamento, melhorar a segurança e a qualidade da assistência prestada ao paciente no PO de CRM e, consequentemente, o desfecho clínico. Sendo assim, o presente estudo tem como objetivo analisar o desempenho de índices de gravidade na predição de complicações em pacientes no PO de revascularização miocárdica durante a permanência na UTI.

## Métodos

Estudo analítico transversal, com dados coletados retrospectivamente por meio da análise de prontuários eletrônicos de pacientes admitidos na UTI cirúrgica de uma instituição especializada em cardiopneumologia de alta complexidade, localizada em São Paulo, Brasil. A pesquisa foi aprovada pelo Comitê de Ética da instituição (Parecer nº 2.831.457).

### Casuística

A casuística, por conveniência, foi composta por pacientes submetidos à CRM entre agosto de 2014 a julho de 2015 e que atenderam aos seguintes critérios de inclusão: idade ≥ 18 anos e ser admitido na UTI diretamente do centro cirúrgico após CRM isolada. Optou-se por incluir pacientes submetidos à CRM exclusiva para evitar a interferência de outros procedimentos cirúrgicos nos desfechos clínicos analisados.

### Variáveis Analisadas

Para atender ao propósito do estudo, foram extraídas dos prontuários dos pacientes as variáveis relacionadas aos dados demográficos (idade, sexo e etnia), à presença dos fatores de risco para doença arterial coronariana (hipertensão arterial sistêmica, diabetes mellitus e dislipidemia) e de IAM prévio, ao procedimento cirúrgico realizado [tipo de cirurgia (eletiva ou de urgência), uso de circulação extracorpórea (CEC), tempo de CEC, tipo de enxerto implantado (arterial, venoso ou misto, ou seja, arterial e venoso) e quantidade média de enxertos recebidos] e à internação na unidade crítica [ocorrência ou não das complicações cardiológicas (arritmia, choque cardiogênico, derrame pericárdico e pericardite), respiratórias (congestão pulmonar, derrame pleural, atelectasia e pneumotórax), neurológicas (delirium, acidente vascular encefálico e crise convulsiva), infecciosas (infecção de ferida operatória, mediastinite, pneumonia, infecção da corrente sanguínea), renais (lesão renal aguda com ou sem terapia de substituição renal, lesão renal crônica agudizada) e hematológicas (sangramento, distúrbios de coagulação, necessidade de transfusão sanguínea), além do tempo de permanência na UTI em dias e da condição de saída da unidade crítica (sobrevivente ou não sobrevivente)]. As complicações analisadas foram selecionadas no estudo por serem as mais frequentes, segundo dados da literatura.^6,8,9^ Ressalta-se que as complicações analisadas na pesquisa foram consideradas presentes a partir do diagnóstico médico das mesmas registrado nos prontuários dos pacientes.

Além dessas variáveis, foram coletadas as informações necessárias para o cálculo dos índices EuroScore,^15^*Acute Physiology and Chronic Health Evaluation* (APACHE II),^16^*Simplified Acute Physiology Score* (SAPS II)^17^ e *Sepsis-related Organ Failure Assessment* (SOFA).^18^ A escolha por esses índices no presente estudo foi pautada na facilidade do cálculo e maior frequência de utilização dos mesmos em estudos^10-14^ que analisam exclusivamente pacientes submetidos à cirurgia cardíaca.

### Procedimento de Coleta dos Dados

Para a coleta de dados, inicialmente foi elaborada uma lista de pacientes submetidos à CRM isolada a partir das descrições cirúrgicas contidas no sistema informatizado da instituição. A partir desta lista, observou-se que todos os pacientes tinham idade superior a 18 anos e foram admitidos na UTI diretamente do centro cirúrgico, não havendo exclusões posteriores.

Para identificar as possíveis complicações ocorridas durante a internação do paciente na UTI após CRM, foram avaliadas todas as evoluções diárias da equipe multiprofissional.

Os dados necessários para o cálculo do risco de morte segundo o EuroScore foram obtidos a partir de informações dos pacientes referentes ao período pré-operatório. Para o cálculo dos índices APACHE II, SAPS II e SOFA foram analisados os sinais vitais e exames laboratoriais das primeiras 24 horas de internação do paciente na UTI, sendo considerados os piores valores, ou seja, que mais pontuavam no índice.

Ressalta-se que a equipe de coleta de dados, formada por enfermeiras que cursavam residência em enfermagem na instituição, foi capacitada por meio de treinamentos in loco. Inicialmente, a coleta de dados foi realizada em pares e qualquer discordância no resgate das informações era prontamente corrigida. Tal processo foi realizado até que se atingisse a uniformidade na coleta de dados entre as enfermeiras.

### Análise Estatística

Para a caracterização da amostra, foram calculadas médias e desvios-padrão para as variáveis contínuas e percentagens absolutas e relativas para as categóricas. Na descrição dos índices de gravidade, média, desvio-padrão, mediana e valores mínimos e máximos foram identificados.

Para avaliar o desempenho dos índices de gravidade na predição das diferentes complicações ocorridas nos pacientes na UTI foram identificados valores de área sob a curva *receiver operating characteristic* (AUC) com respectivo intervalo de confiança (IC) e nível de significância de 95%, ponto de corte ideal calculado pelo método de Youden, sensibilidade, especificidade, valor preditivo positivo (VPP), valor preditivo negativo (VPN) e acurácia. Uma estimativa pontual de AUC ≥ 0,70 foi definida como desempenho satisfatório do índice para o desfecho analisado. O software R versão 3.6.0 para Windows foi utilizado para a análise dos dados.

## Resultados

A casuística foi composta por 366 pacientes, com idade média de 64,58 (± 9,42) anos, predomínio do sexo masculino (75,96%) e etnia caucasiana (88,25%). A presença de hipertensão arterial sistêmica (74,59%) se destacou em relação à dislipidemia (54,10%) e diabetes mellitus (48,63%) como fatores de risco para doença arterial coronariana.

Dos 366 pacientes analisados, aproximadamente metade (n=180; 49,18%) apresentava IAM prévio e a CRM eletiva (60,38%) prevaleceu na amostra. Um total de 295 pacientes (81,69%) foi submetido à CEC durante o procedimento cirúrgico com tempo médio de CEC de 90,69 (± 25,69) minutos. O principal tipo de enxerto implantado foi o misto (80,60%) seguido do arterial (12,84%) e, em média, os pacientes receberam 2,60 (± 0,87) enxertos.

A Tabela 1 apresenta os dados descritivos dos índices de gravidade aplicados na casuística.

**Tabela 1:** – Estatística descritiva de índices de gravidade calculados em pacientes submetidos à CRM

Índice de gravidade	Média	Desvio padrão	Mediana	Mín-Máx
EuroScore*	4,08	4,70	2,50	0,88 - 29,29
SOFA†	5,84	1,88	6,00	2,00 - 13,00
APACHE II‡	14,21	4,96	14,00	4,00 - 41,00
SAPS II§	24,37	8,66	24,00	7,00 - 54,00

*EuroScore: European System for Cardiac Operative Risk Evaluation; †SOFA: Sepsis-related Organ Failure Assessment; ‡APACHE II: Acute Physiology and Chronic Health Evaluation; §SAPS II: Simplified Acute Physiology Score.

O maior risco de morte estimado pelo EuroScore foi de 29,29%. Um total de 9 pacientes tinha chance de morte na UTI acima de 50% conforme previsto pelo APACHE II, 2 segundo SOFA e 1 de acordo com o SAPS II.

Na amostra de 366 pacientes, as frequências das complicações evidenciadas no PO de CRM na UTI foram: respiratórias (24,32%), cardiológicas (19,95%), neurológicas (10,38%), hematológicas (10,38%), infecciosas (6,56%) e renais (3,55%).

Entre elas, destacaram-se a congestão pulmonar (n=52) e o derrame pleural (n=30) para complicações respiratórias, arritmias (n=55) nas cardiológicas, delirium (n=34) nas neurológicas e pneumonia (n=14) para as infecciosas. A lesão renal aguda e a hemorragia representaram a totalidade das complicações renais e hematológicas, respectivamente.

O tempo de permanência dos pacientes na UTI foi 4,64 (± 5,64) dias e todos os doentes que evoluíram a óbito na unidade crítica (n=19; 5,20%) apresentaram algum tipo de complicação no PO de CRM.

**Figura 1:**
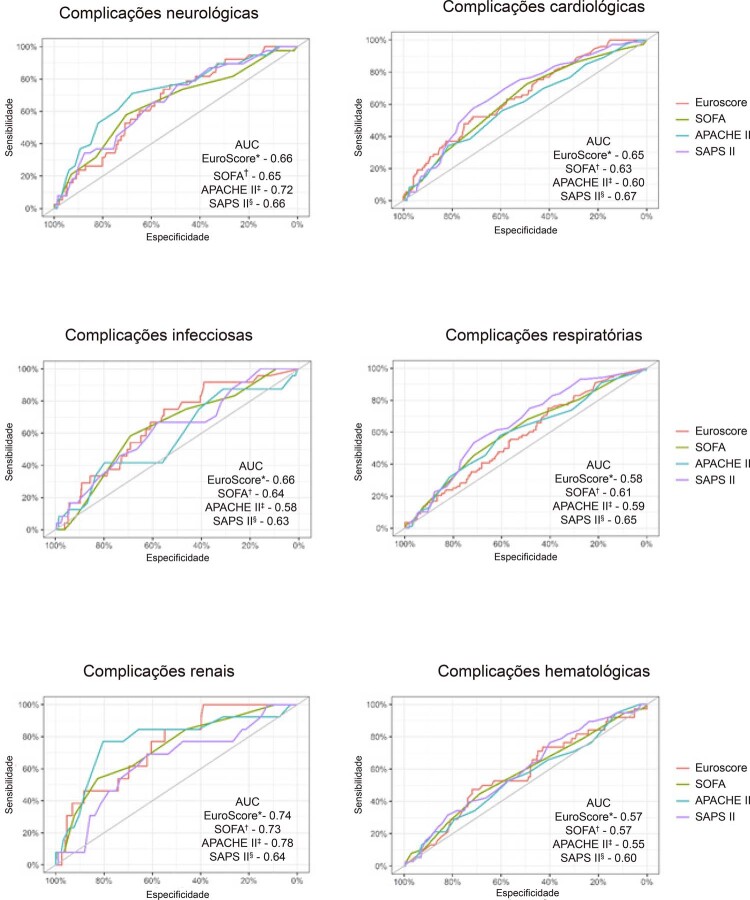
– Receiver Operating Characteristic Curves dos índices de gravidade na predição de complicações em pacientes após CRM. *EuroScore: European System for Cardiac Operative Risk Evaluation; †SOFA: Sepsis-related Organ Failure Assessment; ‡APACHE II: Acute Physiology and Chronic Health Evaluation; §SAPS II: Simplified Acute Physiology Score.

A partir dos dados da Figura 1 e Tabela 2, identificou-se que o APACHE II foi o índice que apresentou melhor desempenho na predição de complicações neurológicas e renais apresentadas pelos pacientes no PO de CRM durante a internação na UTI, com maiores valores de AUC e resultados satisfatórios na análise dos valores de sensibilidade, especificidade, VPN e acurácia em comparação aos outros índices. Em contrapartida, nenhum dos índices aplicados (EuroScore, SOFA, APACHE II e SAPS II) apresentou bom desempenho na predição das outras complicações (cardiológicas, infecciosas, respiratórias e hematológicas) investigadas no estudo.

**Tabela 2:** – Desempenho dos índices de gravidade na predição de complicações em pacientes no pós-operatório de CRM na UTI

Neurológicas	AUC^//^	IC95%^¶^ mín-máx	Ponto de corte	Sensibilidade	Especificidade	VPP^#^	VPN**	Acurácia
EuroScore*	0,66	0,57-0,74	2,59	73,68	55,05	16,00	94,74	56,99
SOFA^†^	0,65	0,55-0,74	6,50	57,89	70,34	18,49	93,50	69,04
APACHE II^‡^	0,72	0,63-0,81	15,50	71,05	67,89	20,45	95,28	68,22
SAPS II^§^	0,66	0,57-0,75	23,50	76,32	49,24	14,87	94,71	52,05
**Cardiológicas**	**AUC^//^**	**IC95%^¶^ mín-máx**	**Ponto de corte**	**Sensibilidade**	**Especificidade**	**VPP^#^**	VPN**	**Acurácia**
EuroScore*	0,65	0,58-0,72	3,50	52,05	71,58	31,40	85,66	67,67
SOFA^†^	0,63	0,56-0,70	5,50	72,60	49,32	26,37	87,80	53,97
APACHE II^‡^	0,60	0,52-0,67	17,50	34,25	81,51	31,65	83,22	72,05
SAPS II^§^	0,67	0,60-0,74	27,50	57,53	71,23	33,33	87,03	68,49
**Infecciosas**	**AUC^//^**	**IC95%^¶^ mín-máx**	**Ponto de corte**	**Sensibilidade**	**Especificidade**	**VPP^#^**	VPN**	**Acurácia**
EuroScore*	0,66	0,56-0,78	1,92	91,67	39,00	9,57	98,52	42,47
SOFA^†^	0,64	0,52-0,74	6,50	58,33	69,21	11,76	95,93	68,49
APACHE II^‡^	0,58	0,46-0,70	17,50	41,67	79,77	12,66	95,10	77,26
SAPS II^§^	0,63	0,53-0,74	25,50	66,67	58,06	10,06	96,12	58,63
**Respiratórias**	**AUC^//^**	**IC95%^¶^ mín-máx**	**Ponto de corte**	**Sensibilidade**	**Especificidade**	**VPP^#^**	VPN**	**Acurácia**
EuroScore*	0,58	0,51-0,64	1,92	75,00	40,79	28,70	83,70	49,04
SOFA^†^	0,61	0,54-0,67	5,50	68,18	49,10	29,85	82,93	53,70
APACHE II^‡^	0,59	0,52-0,66	14,50	57,95	60,65	31,87	81,95	60,00
SAPS II^§^	0,65	0,58-0,71	27,50	53,41	71,48	37,30	82,85	67,12
**Renais**	**AUC^//^**	**IC95%^¶^ mín-máx**	**Ponto de corte**	**Sensibilidade**	**Especificidade**	**VPP^#^**	VPN**	**Acurácia**
EuroScore*	0,74	0,62-0,86	2,65	84,62	54,83	6,47	98,97	55,89
SOFA^†^	0,73	0,59-0,87	7,50	53,85	82,67	10,29	97,98	81,64
APACHE II^‡^	0,78	0,63-0,93	17,50	76,92	80,40	12,66	98,95	80,27
SAPS II^§^	0,64	0,48-0,80	26,50	69,23	61,65	6,25	98,19	61,92
**Hematológicas**	**AUC^//^**	**IC95%^¶^ mín-máx**	**Ponto de corte**	**Sensibilidade**	**Especificidade**	**VPP^#^**	VPN**	**Acurácia**
EuroScore*	0,57	0,48-0,67	1,52	71,87	47,37	92,16	16,36	69,32
SOFA^†^	0,57	0,47-0,67	6,50	44,74	68,81	14,29	91,46	66,30
APACHE II^‡^	0,55	0,45-0,65	14,50	52,63	57,19	12,50	91,22	56,71
SAPS II^§^	0,60	0,50-0,68	21,50	76,32	40,06	12,89	93,57	43,84

*EuroScore: European System for Cardiac Operative Risk Evaluation; ^†^SOFA: Sepsis-related Organ Failure Assessment; ^‡^APACHE II: Acute Physiology and Chronic Health Evaluation; ^§^SAPS II: Simplified Acute Physiology Score; ^//^AUC: área sob a curva; ^¶^IC: intervalo de confiança; ^#^VPP: valor preditivo positivo; **VPN: valor preditivo negativo.

## Discussão

Os dados da casuística deste estudo mostraram predomínio do sexo masculino e idade média aproximada de 64 anos, corroborando com achados na literatura.^10,11^

Na análise dos índices de gravidade, identificou-se que o valor médio do EuroScore encontrado neste estudo foi superior aos resultados de pesquisas que aplicaram o índice em pacientes após cirurgia cardíaca^11,14^ ou CRM isolada.^10^Entretanto, os valores do SOFA, APACHE II e SAPS II desta investigação foram inferiores aos achados da pesquisa que analisou 150 pacientes submetidos à cirurgia cardíaca e comparou o desempenho desses índices e do *Cardiac Surgery Score* (CASUS) na predição de desfechos clínicos, com resultado satisfatório para o CASUS.^11^

As principais complicações apresentadas pelos pacientes no período PO da CRM na UTI corroboram com dados da literatura.^7-9^ Distúrbios respiratórios são comuns em pacientes após cirurgia cardíaca, mas poucos necessitam de ventilação mecânica por mais de 72 horas para tratamento.^19,20^Arritmias supraventriculares, especialmente fibrilação atrial (FA), ocorrem com frequência após cirurgia cardíaca e contribuem para o aumento de tempo de internação hospitalar e risco de acidente vascular encefálico, sendo que a terapia profilática diminui a incidência de FA em 50%.7

Interessante observar o número de pacientes que apresentaram delirium na presente investigação. Estudos mostram que este evento é um problema significativo após cirurgia cardíaca, com impacto negativo no desfecho clínico dos pacientes,^21-23^ e os principais fatores de risco são uso de benzodiazepínico, restrições e imobilizações no leito devido à presença de dispositivos.^24^ Além disso, deve-se ressaltar que 247 pacientes da amostra tinham mais de 60 anos (idade média aproximada de 64 anos) e revisão da literatura^25^evidenciou que existe associação entre idade avançada e maior risco de delirium na UTI.

As infecções nosocomiais ocorrem em 10 a 20% dos pacientes submetidos à cirurgia cardíaca e muitas delas são evitáveis.^7^ Por fim, destacam-se a lesão renal aguda e a hemorragia. A primeira decorre principalmente de hipoperfusão, hemólise e citocinas inflamatórias e, quando é necessária a terapia de substituição renal, há aumento significativo de mortalidade.^7,26^ A hemorragia excessiva, identificada habitualmente pelas características e volume do débito nos drenos de tórax, ocorre principalmente após cirurgias de emergência, uso prolongado da CEC, baixa massa corporal e anemia pré-operatória.^7^

Em relação à aplicabilidade dos índices de gravidade, este estudo identificou que apenas o APACHE II apresentou satisfatório desempenho na predição das complicações neurológicas e renais apresentadas pelos pacientes no pós operatório de CRM durante a internação na UTI. Para os outros desfechos analisados, nenhum índice aplicado na casuística foi considerado bom preditor.

Estudos recentes mostram que o APACHE II é considerado um adequado índice preditivo de complicações neurológicas em pacientes cardíacos comatosos após parada cardiorrespiratória^27^ e uma excelente ferramenta prognóstica para mortalidade em pacientes com lesão renal aguda.^28^ Entretanto, essas pesquisas, apesar de analisarem complicações neurológicas e renais ocorridas em UTI, não abordaram especificamente pacientes submetidos à CRM isolada.

Vale ressaltar que o APACHE II é um índice reprodutível, de fácil aplicação, que fornece a probabilidade de morte do paciente a partir de diferentes informações sobre fatores que influenciam este desfecho, como idade, presença de comorbidades e alterações fisiológicas. Uma limitação deste índice é que alguns pacientes apresentam várias comorbidades e apenas uma delas pode ser selecionada.^29^ No contexto cardiovascular, somente a insuficiência cardíaca congestiva classe IV é valorizada no seu cálculo.^16^

Em relação ao EuroScore, estudo brasileiro mostrou valores de AUC > 0,70 na predição de complicações renais e respiratórias após cirurgia cardíaca, não especificamente em pacientes submetidos à CRM isolada.^14^ Neste sentido, deve ser reforçado que o EuroScore é um índice específico para avaliação de risco em diferentes cirurgias cardíacas, incluindo a CRM, e considera, para seu cálculo, dados pré-operatórios.^15^ Portanto, supõe-se que estas características do índice tenham influenciado para seu baixo desempenho na predição de complicações dos pacientes após CRM isolada do presente estudo.

O SAPS II, em seu estudo de validação, não incluiu pacientes de cirurgia cardíaca.^17^ Acredita-se que esta seja a principal causa do inadequado desempenho do índice nos desfechos analisados.

Por fim, o SOFA^18^ foi criado para identificar disfunção orgânica, que nem sempre está presente nas primeiras 24 horas de internação na UTI. Neste sentido, resultados de pesquisa sugerem que o índice seja calculado sequencialmente, uma vez que, independentemente do valor inicialmente encontrado, o aumento do escore nas primeiras 48 horas do paciente na unidade crítica prediz uma taxa de mortalidade de, no mínimo, 50%.^30^ Este fato pode ter interferido nos achados do presente estudo, uma vez que o SOFA foi calculado apenas com dados das primeiras 24 horas do paciente na UTI e foram consideradas as complicações ocorridas durante toda a internação na terapia intensiva.

Finalizando, destaca-se que esta pesquisa foi pioneira na avaliação do desempenho de diferentes índices de gravidade, habitualmente aplicados em doentes críticos, na predição de complicações em uma população específica: pacientes submetidos à CRM isolada.

Este estudo apresenta as seguintes limitações: a análise documental retrospectiva dependente da precisão e qualidade das informações registradas pelos profissionais, a casuística foi limitada e o estudo realizado em um único hospital, centro de referência em cardiologia, trazendo possíveis restrições na generalização dos resultados.

## Conclusões

Este estudo permitiu concluir que o índice APACHE II apresentou satisfatório desempenho na predição de complicações neurológicas e renais apresentadas pelos pacientes submetidos à CRM isolada durante a internação na UTI. Em relação à ocorrência de complicações cardiológicas, infecciosas, respiratórias e hematológicas, nenhum dos índices analisados (EuroScore, SOFA, APACHE II e SAPS II) apresentaram bons resultados preditivos.

Portanto, os índices analisados no estudo não devem ser utilizados indiscriminadamente com o objetivo de predizer de maneira precoce as complicações mais frequentes apresentadas por pacientes após CRM na UTI e sugere-se a realização de estudos multicêntricos, com amostras significativas, que proponham a criação de índices específicos para prever essas complicações.
