# Supplementary material for: Desempenho dos Índices de Gravidade na Predição de Complicações Pós-Operatórias de Revascularização Miocárdica
Source: Arq Bras Cardiol. 2020 Sep 18;115(3):452–9. [Article in Portuguese] doi: 10.36660/abc.20190120 (PMC9363080; doi:10.36660/abc.20190120)
Supplement: Translation

# Translation: Performance of Severity Indices to Estimate Postoperative Complications of Myocardial Revascularization

**Authors:** Silvana Alves dos Santos Franzotti, Dyenily Alessi Sloboda, Juliana Rosendo Silva, Ellian Amorim Santos Souza, Jessica Zamora Reboreda, Renata Eloah de Lucena Ferretti-Rebustini, Lilia de Souza Nogueira

**Affiliations:** 1 Brazil Universidade de São Paulo - Escola de Enfermagem, São Paulo, SP - Brazil; 2 Brazil Universidade de São Paulo Faculdade de Medicina Hospital das Clínicas Instituto do Coração, São Paulo, SP – Brazil

**Keywords:** Cardiovascular Diseases/complications, Cardiovascular Diseases/surgery, Myocardial Revascularization/complications, Postoperative Care, Indicators of Morbidity and Mortality, Severity of Illness Index, Intensive Care Units

## Abstract

**Background:**

Patients in the postoperative period of myocardial revascularization (Coronary Artery Bypass Grafting - CABG) surgery admitted to the intensive care unit (ICU) are at risk of complications which increase the length of stay and morbidity and mortality. Therefore, early recognition of these risks is essential to optimize prevention strategies and a satisfactory clinical outcome.

**Objective:**

To analyze the performance of severity indices in predicting complications in patients in the postoperative of CABG during the ICU stay.

**Methods:**

A cross-sectional study with retrospective analysis of electronic medical records of patients aged ≥ 18 years who underwent isolated CABG and were admitted to the ICU of a cardiology hospital in São Paulo, Brazil. The areas under the receiver operating characteristic curves (AUC) with a 95% confidence interval were analyzed to verify the accuracy of the European System for Cardiac Operative Risk Evaluation (EuroScore), Acute Physiology and Chronic Health Evaluation (APACHE II), Simplified Acute Physiology Score (SAPS II) and Sequential Organ Failure Assessment (SOFA) indices in predicting complications.

**Results:**

The sample consisted of 366 patients (64.58 ± 9.42 years; 75.96% male). The complications identified were: respiratory (24.32%), cardiovascular (19.95%), neurological (10.38%), hematological (10.38%), infectious (6.56%) and renal (3.55%). APACHE II showed satisfactory performance for predicting neurological (AUC 0.72) and renal (AUC 0.78) complications.

**Conclusion:**

APACHE II excelled in predicting neurological and renal complications. None of the indices performed well in predicting the other analyzed complications. Therefore, severity indices should not be used indiscriminately in order to predict all complications frequently presented by patients after CABG. (Arq Bras Cardiol. 2020; 115(3):452-459)

## Introduction

Cardiovascular diseases are responsible for a high mortality rate in Brazil and in the world, with emphasis on the high prevalence of acute myocardial infarction (MI).^1-3^ Myocardial revascularization (Coronary Artery Bypass Grafting - CABG) surgery is among the main therapies indicated for treating acute MI.^4,5^

Successful results of CABG depend on excellent postoperative care performed in the intensive care unit (ICU), since patients are exposed to numerous adverse effects resulting from the complex organic systemic inflammatory response, present hemodynamic instability and greater risk of developing complications during intensive treatment.^6-9^

Early recognition of patients at high risk of complications is essential to optimize treatment, increase the chance of a satisfactory outcome,^6-9^in addition to reduce costs since the occurrence of these events after CABG significantly increases treatment and hospitalization expenses.^10^

Recent studies have shown the excellent accuracy of different severity indices in predicting mortality of patients in the postoperative period of cardiac surgery in the ICU.^11-13^ Regarding the occurrence of complications, researchers identified that the European System for Cardiac Operative Risk Evaluation index (EuroScore) was a good predictor of respiratory failure and acute kidney injury with renal replacement therapy in patients after cardiac surgery.^14^

In view of the above, until the present moment there are no specific indices which are capable of satisfactorily predicting the risk of a patient developing different complications in the ICU after CABG. In addition, intensive care professionals in clinical practice identify a close relationship between patient severity in the postoperative period of CABG and the occurrence of complications.

In this sense, the importance of analyzing and investigating the performance of different severity indices in predicting these complications is reinforced, since this information may assist in treatment strategies, improve safety and the quality of care provided to patients in the postoperative period of CABG, and consequently the clinical outcome. Therefore, the present study aims to analyze the performance of severity indices in predicting complications in patients in the postoperative of CABG during their stay in the ICU.

## Methods

A cross-sectional analytical study with data retrospectively collected through an analysis of electronic medical records of patients admitted to the surgical ICU of an institution specialized in highly complex cardiopneumology, located in São Paulo, Brazil. The study was approved by the institution’s Ethics Committee (Opinion no. 2,831,457).

### Sample

The convenience sample consisted of patients who underwent CABG between August 2014 and July 2015 and who met the following inclusion criteria: age ≥ 18 years and being admitted to the ICU directly from the operating room after isolated CABG. We chose to include patients undergoing exclusive CABG to avoid interference from other surgical procedures in the analyzed clinical outcomes.

### Analyzed Variables

Variables related to demographic data (age, gender and ethnicity), the presence of risk factors for coronary artery disease (systemic arterial hypertension, diabetes mellitus and dyslipidemia) and previous acute MI, surgical procedure performed [type of surgery (elective or emergency), use of cardiopulmonary bypass (CPB), duration of CPB, type of graft implanted (arterial, venous or mixed, meaning arterial and venous) and average amount of grafts received] and admission to the critical unit [occurrence or not of cardiovascular (arrhythmia, cardiogenic shock, pericardial effusion and pericarditis), respiratory (pulmonary congestion, pleural effusion, atelectasis and pneumothorax), neurological (delirium, stroke and seizure), infectious (wound infection, mediastinitis, pneumonia, bloodstream infection), kidney (acute kidney injury with or without renal replacement therapy, acute chronic kidney injury) or hematological complications (bleeding, coagulation disorders, need for blood transfusion), in addition to the length of stay in the ICU in days and the condition of critical unit discharge (surviving or not surviving)] were extracted from the patients’ medical records. The analyzed complications were selected in the study because they are the most frequent according to data in the literature.^6,8,9^ It is noteworthy that the complications analyzed in the study were considered present based on their medical diagnosis recorded in the patients’ medical records.

In addition to these variables, the necessary information was collected to calculate the EuroScore,^15^ Acute Physiology and Chronic Health Evaluation (APACHE II),^16^ Simplified Acute Physiology Score (SAPS II)^17^ and the Sepsis-related Organ Failure Assessment (SOFA).^18^ The choice of these indices in the present study was based on the ease of calculation and their higher frequency of use in studies^10-14^which exclusively analyze patients undergoing cardiac surgery.

### Data Collection Procedure

A list of patients undergoing CABG isolated from the surgical descriptions contained in the institution’s computerized system was initially created for data collection. From this list, it was observed that all patients were over 18 years old and were admitted to the ICU directly from the operating room, with no subsequent exclusions.

All the daily evolutions of the multidisciplinary team were evaluated in order to identify the possible complications which occurred during the patient’s stay in the ICU after CABG.

The data needed to calculate the risk of death according to the EuroScore were obtained from the patient information regarding the preoperative period. The vital signs and laboratory tests of the first 24 hours of the patient’s stay in the ICU were analyzed to calculate the APACHE II, SAPS II and SOFA indices, with those which scored the highest on the index being considered the worst values.

It is noteworthy that the data collection team was formed by residency nurses at the institution and trained on-site. The data collection was initially performed in pairs, and any disagreement in the information retrieval was promptly corrected. This process was carried out until uniformity in data collection among nurses was achieved.

### Statistical Analysis

Means and standard deviations were calculated for continuous variables and absolute and relative percentages for categorical variables in order to characterize the sample. Mean, standard deviation, median and minimum and maximum values were identified in the severity indices description.

The area under the receiver operating characteristic curve (AUC) values with respective confidence interval (CI) and 95% significance level, the ideal cut-off point calculated by the Youden method, sensitivity, specificity, positive predictive value (PPV), negative predictive value (NPV) and accuracy were identified to assess the performance of the severity indices in predicting the different complications which occurred in patients in the ICU. A point estimate of AUC ≥ 0.70 was defined as satisfactory performance of the index for the analyzed outcome. The R version 3.6.0 software program for Windows was used for data analysis.

## Results

The sample consisted of 366 patients with a mean age of 64.58 (± 9.42) years, predominantly male (75.96%) and Caucasian (88.25%). The presence of systemic arterial hypertension (74.59%) stood out in relation to dyslipidemia (54.10%), and diabetes mellitus (48.63%) as risk factors for coronary artery disease.

Of the 366 patients analyzed, approximately half (n = 180; 49.18%) had previous acute MI and elective CABG (60.38%) prevailed in the sample. A total of 295 patients (81.69%) underwent CPB during the surgical procedure with an average CPB time of 90.69 (± 25.69) minutes. The main type of implanted graft was mixed (80.60%), followed by arterial (12.84%), and patients received an average of 2.60 (± 0.87) grafts.

Table 1 presents the descriptive data of the severity indices applied in the sample.

**Table 1:** – Descriptive statistics of severity indices calculated in patients undergoing CABG

Severity index	Mean	Standard deviation	Median	Min-Max
EuroScore*	4.08	4.70	2.50	0.88 - 29.29
SOFA†	5.84	1.88	6.00	2.00 - 13.00
APACHE II‡	14.21	4.96	14.00	4.00 - 41.00
SAPS II§	24.37	8.66	24.00	7.00 - 54.00

*EuroScore: European System for Cardiac Operative Risk Evaluation; †SOFA: Sepsis-related Organ Failure Assessment; ‡APACHE II: Acute Physiology and Chronic Health Evaluation; §SAPS II: Simplified Acute Physiology Score.

The highest risk of death estimated by EuroScore was 29.29%. A total of 9 patients had more than 50% of chance of death in the ICU as predicted by APACHE II, 2 according to SOFA and 1 according to SAPS II.

The frequencies of complications evidenced in the postoperative period of CABG in the ICU in the sample of 366 patients were: respiratory (24.32%), cardiovascular (19.95%), neurological (10.38%), hematological (10.38%), infectious (6.56%), and renal (3.55%).

Among these, pulmonary congestion (n = 52) and pleural effusion (n = 30) stood out for respiratory complications, arrhythmias (n = 55) in cardiovascular, delirium (n = 34) in neurological and pneumonia (n = 14) for infectious. Acute kidney injury and hemorrhaging accounted for all kidney and hematological complications, respectively.

The length of stay of patients in the ICU was 4.64 (± 5.64) days, and all patients who died in the critical unit (n = 19; 5.20%) presented some type of complication in the CABG postoperative period.

From the data in Figure 1 and Table 2, it was identified that APACHE II was the index which had the best performance in predicting neurological and renal complications presented by patients in the postoperative of CABG during ICU admission, along with higher AUC values and satisfactory results in the analysis of sensitivity, specificity, NPV and accuracy values compared to other indexes. In contrast, none of the applied indices (EuroScore, SOFA, APACHE II and SAPS II) performed well in predicting other complications (cardiovascular, infectious, respiratory and hematological) investigated in the study.

**Figure 1:** – Receiver Operating Characteristic Curves of severity indices in predicting complications in patients after CABG. *EuroScore: European System for Cardiac Operative Risk Evaluation; †SOFA: Sepsis-related Organ Failure Assessment; ‡APACHE II: Acute Physiology and Chronic Health Evaluation; §SAPS II: Simplified Acute Physiology Score.

**Table 2:** – Performance of severity indices in predicting complications in patients in the postoperative period of CABG in the ICU

Neurological	AUC^//^	95%CI^¶^ min-max	Cut-off point	Sensitivity	Specificity	PPV^#^	NPV^**^	Accuracy
EuroScore*	0.66	0.57-0.74	2.59	73.68	55.05	16.00	94.74	56.99
SOFA^†^	0.65	0.55-0.74	6.50	57.89	70.34	18.49	93.50	69.04
APACHE II^‡^	0.72	0.63-0.81	15.50	71.05	67.89	20.45	95.28	68.22
SAPS II^§^	0.66	0.57-0.75	23.50	76.32	49.24	14.87	94.71	52.05
**Cardiovascular**	**AUC//**	**95%CI^¶^ min-max**	**Cut-off point**	**Sensitivity**	**Specificity**	**PPV^#^**	NPV**	**Accuracy**
EuroScore*	0.65	0.58-0.72	3.50	52.05	71.58	31.40	85.66	67.67
SOFA^†^	0.63	0.56-0.70	5.50	72.60	49.32	26.37	87.80	53.97
APACHE II^‡^	0.60	0.52-0.67	17.50	34.25	81.51	31.65	83.22	72.05
SAPS II^§^	0.67	0.60-0.74	27.50	57.53	71.23	33.33	87.03	68.49
**Infectious**	**AUC^//^**	**95%CI^¶^ min-max**	**Cut-off point**	**Sensitivity**	**Specificity**	**PPV^#^**	NPV**	**Accuracy**
EuroScore*	0.66	0.56-0.78	1.92	91.67	39.00	9.57	98.52	42.47
SOFA^†^	0.64	0.52-0.74	6.50	58.33	69.21	11.76	95.93	68.49
APACHE II^‡^	0.58	0.46-0.70	17.50	41.67	79.77	12.66	95.10	77.26
SAPS II^§^	0.63	0.53-0.74	25.50	66.67	58.06	10.06	96.12	58.63
**Respiratory**	**AUC^//^**	**95%CI^¶^ min-max**	**Cut-off point**	**Sensitivity**	**Specificity**	**PPV^#^**	NPV**	**Accuracy**
EuroScore*	0.58	0.51-0.64	1.92	75.00	40.79	28.70	83.70	49.04
SOFA^†^	0.61	0.54-0.67	5.50	68.18	49.10	29.85	82.93	53.70
APACHE II^‡^	0.59	0.52-0.66	14.50	57.95	60.65	31.87	81.95	60.00
SAPS II^§^	0.65	0.58-0.71	27.50	53.41	71.48	37.30	82.85	67.12
**Renal**	**AUC^//^**	**95%CI^¶^ min-max**	**Cut-off point**	**Sensitivity**	**Specificity**	**PPV^#^**	NPV**	**Accuracy**
EuroScore*	0.74	0.62-0.86	2.65	84.62	54.83	6.47	98.97	55.89
SOFA^†^	0.73	0.59-0.87	7.50	53.85	82.67	10.29	97.98	81.64
APACHE II^‡^	0.78	0.63-0.93	17.50	76.92	80.40	12.66	98.95	80.27
SAPS II^§^	0.64	0.48-0.80	26.50	69.23	61.65	6.25	98.19	61.92
**Hematological**	**AUC^//^**	**95%CI^¶^ min-max**	**Cut-off point**	**Sensitivity**	**Specificity**	**PPV^#^**	NPV**	**Accuracy**
EuroScore*	0.57	0.48-0.67	1.52	71.87	47.37	92.16	16.36	69.32
SOFA^†^	0.57	0.47-0.67	6.50	44.74	68.81	14.29	91.46	66.30
APACHE II^‡^	0.55	0.45-0.65	14.50	52.63	57.19	12.50	91.22	56.71
SAPS II^§^	0.60	0.50-0.68	21.50	76.32	40.06	12.89	93.57	43.84

*EuroScore: European System for Cardiac Operative Risk Evaluation; ^†^SOFA:- Sepsis-related Organ Failure Assessment; ^‡^APACHE II: Acute Physiology and Chronic Health Evaluation; ^§^SAPS II: Simplified Acute Physiology Score; ^//^AUC: Area under the curve; ^¶^CI: Confidence interval; ^#^PPV: positive predictive value; **NPV: Negative predictive value.

## Discussion

The sample data in this study showed a predominance of males and an average age of approximately 64 years, corroborating findings in the literature.^10,11^In analyzing the severity indices, it was found that the average EuroScore value found in this study was higher than the results of a study which applied the index to patients after cardiac surgery^11,14^or isolated CABG.^10^ However, the SOFA, APACHE II and SAPS II values of this investigation were inferior to the findings of a study which analyzed 150 patients undergoing cardiac surgery and compared the performance of these indexes and the Cardiac Surgery Score (CASUS) in predicting clinical outcomes, with a satisfactory result for CASUS.^11^

The main complications presented by patients in the CABG postoperative period in the ICU corroborate with data from the literature.^7-9^ Respiratory disorders are common in patients after cardiac surgery, but few require mechanical ventilation for more than 72 hours for treatment.^19,20^ Supraventricular arrhythmias, especially atrial fibrillation (AF), frequently occur after cardiac surgery and contribute to increased hospital stay and risk of stroke, with prophylactic therapy decreasing the incidence of AF by 50%.^7^

It is interesting to note the number of patients who had delirium in the present investigation. Studies show that this event is a significant problem after cardiac surgery, with a negative impact on the clinical outcome of patients,^21-23^ and the main risk factors being benzodiazepine use, restrictions and bed immobilizations due to the presence of devices.^24^In addition, it should be noted that 247 patients in the sample were older than 60 years (mean age approximately 64 years) and a literature review^25^ showed that there is an association between advanced age and a higher risk of delirium in the ICU.

Nosocomial infections occur in 10 to 20% of patients undergoing cardiac surgery and many of them are preventable.^7^ Finally, acute kidney injury and hemorrhaging stand out. The first is mainly due to hypoperfusion, hemolysis and inflammatory cytokines, and there is a significant increase in mortality when renal replacement therapy is necessary.^7,26^ Excessive hemorrhaging is usually identified by the characteristics and volume of the output in the chest drains, and mainly occurs after emergency surgery, prolonged use of CPB, low body mass and preoperative anemia.^7^

Regarding the applicability of severity indices, this study identified that only that APACHE II had a satisfactory performance in predicting neurological and renal complications presented by patients in the postoperative period of CABG during ICU admission. No other index applied in the sample was considered a good predictor for the other analyzed outcomes.

Recent studies show that APACHE II is considered an adequate predictive index of neurological complications in comatose cardiac patients after cardiac arrest^27^ and an excellent prognostic tool for mortality in patients with acute kidney injury.^28^ However, despite analyzing neurological and renal complications occurring in the ICU, these studies did not specifically address patients undergoing isolated CABG.

It is worth mentioning that APACHE II is a reproducible and easy-to-apply index which provides the patient’s probability of death based on different information of factors which influence this outcome such as age, the presence of comorbidities and physiological changes. A limitation of this index is that some patients have several comorbidities and only one of them can be selected.^29^ In the cardiovascular context, only class IV congestive heart failure is valued in its calculation.^16^

Regarding the EuroScore, a Brazilian study showed AUC values > 0.70 in predicting renal and respiratory complications after cardiac surgery, not specifically in patients undergoing isolated CABG.^14^ In this sense, it must be reinforced that the EuroScore is a specific index for risk assessment in different cardiac surgeries, including CABG, and considers preoperative data for its calculation.^15^ Therefore, it is assumed that these characteristics of the index influenced its low performance in predicting complications in patients after isolated CABG in the present study.

The SAPS II validation study did not include cardiac surgery patients.^17^ This is believed to be the main cause of the inadequate performance of the index in the analyzed outcomes.

Lastly, the SOFA^18^ was created to identify organ dysfunction, which is not always present in the first 24 hours of admission to the ICU. In this sense, research results suggest that the index should be calculated sequentially, since the increase in the score in the first 48 hours of the patient in the ICU predicts a mortality rate of at least 50%, regardless of the value initially found.^30^ This fact may have interfered in the findings of the present study, since the SOFA was only calculated with data from the patient’s first 24 hours in the ICU, and the complications which occurred during the entire intensive care stay were considered.

Finally, it should be noted that this study was a pioneer in evaluating the performance of different severity indices usually applied to critically ill patients in predicting complications in a specific population: patients undergoing isolated CABG.

This study has the following limitations: the retrospective document analysis was dependent on the accuracy and quality of the information recorded by the professionals, the sample was limited, and the study carried out in a single hospital (a reference cardiology center), leading to possible restrictions on the generalization of the results.

## Conclusions

This study enables concluding that the APACHE II index showed a satisfactory performance in predicting neurological and renal complications presented by patients submitted to isolated CABG during ICU admission. None of the analyzed indexes (EuroScore, SOFA, APACHE II and SAPS II) showed good predictive results regarding the occurrence of cardiovascular, infectious, respiratory or hematological complications.

Therefore, the indexes analyzed in the study should not be used indiscriminately in order to predict the most frequent complications presented by patients after CABG in the ICU early, and it is suggested that multicenter studies be conducted with significant samples which propose creating specific indices to predict these complications.
